# The role of SOCS3 in the hypothalamic paraventricular nucleus in rat model of inflammatory pain

**DOI:** 10.1186/s12950-020-00241-9

**Published:** 2020-02-27

**Authors:** Na Meng, Ning-Ning Ji, Ziming Zhou, Yicheng Qian, Yu Tang, Kangbo Yang, Binbin Chen, Yong-Mei Zhang

**Affiliations:** Jiangsu Province Key Laboratory of Anesthesiology, Xuzhou Medical University, Xuzhou, 221002 Jiangsu China

**Keywords:** SOCS, IL-6, PVN, Inflammatory pain, CFA

## Abstract

**Background:**

Inflammatory molecular signals are modulated by a variety of intracellular transduction pathways, the activation of which may induce and amplify the spread of inflammatory response. Suppresser of cytokine signaling 3 (SOCS3) is an established negative feedback regulation transcription factor associated with tumor, diabetes mellitus, inflammation and anaphylaxis. Herein, we investigated whether SOCS3 in the paraventricular nucleus (PVN) can attenuate pro-inflammatory responses, and thereby relieve the inflammatory pain.

**Methods:**

Adeno-associated virus (AAV) overexpressing SOCS3 was pre-injected into the PVN. Three weeks later, rat model of chronic inflammatory pain was established via subcutaneous injection of complete Freund’s adjuvant (CFA) into the plantar center of hind paws. The therapeutic effect of SOCS3 was tested by the measurement of thermal and mechanical allodynia. In mechanistic study, the protein level of SOCS3 was evaluated by Western blotting, and the expression of c-fos and Iba-1 were assessed by immunofluorescent staining.

**Results:**

Inflammatory pain was associated with upregulated interleukin 6 (IL-6) and SOCS3 in PVN in the acute phase. Thermal hyperalgesia can be relieved by intra-PVN injection of IL-6 neutralizing antibody (NA). Meanwhile, the upregulated c-fos and microglial activation was reversed. Furthermore, SOCS3 expression in PVN was downregulated in the chronic phase. Intra-PVN injection of AAV overexpressing SOCS3 suppressed the activation of neurons and attenuated thermal hyperalgesia and mechanical allodynia.

**Conclusion:**

Inhibition of IL-6 signaling attenuated inflammatory hyperalgesia in the acute phase. SOCS3 overexpression in the PVN attenuated inflammatory pain in the chronic phase via suppression of neuronal activation.

## Background

Hypothalamic paraventricular nucleus (PVN) is an autonomic control center involved in immunological stress and neuroendocrine regulation [1], which can receive and integrate multiple input signals, and thereafter generate complex multifactorial autonomic output by concurrent modification of the excitability of multiple output pathways [2]. Glial cells are resident immune effector cells in the central nervous system (CNS), and are involved in modulation of neuronal synaptic and neuronal excitability via a wealth of mechanisms [3]. On activation, glial cells release inflammatory mediators to exert both beneficial and detrimental effects on CNS [4]. Prior studies demonstrated that the activated corticotropin-releasing factor (CRF) neurons and microglia in PVN promoted the secretion of pro-inflammatory factors, such as interleukin 1β (IL-1β), IL-6 and tumor necrosis factor α (TNF-α), which precipitated the colorectal distension-induced visceral hypersensitivity in rats [5].

Inflammatory pain, characterized by spontaneous pain, hyperalgesia and allodynia, mainly results from trauma, pathogen infection, acidosis or other diseases [6, 7]. To attenuate inflammation, the intracellular negative regulator suppressor of cytokine signaling (SOCS) proteins are secreted to inhibit pro-inflammatory cytokine signals [8]. The potency of SOCS has been reported in the treatment of inflammatory arthritis, hematological malignancies and infectious disease [9–11]. As a significant member of the SOCS family, SOCS3 can modulate the innate and adaptive immune responses by regulating the extent and duration of cytokine-induced signaling. In exposure to noxious stimulus, SOCS3 is rapidly secreted to inhibit STAT3 phosphorylation to block IL-6-mediated inflammatory signaling. Notably, SOCS3 can be rapidly degraded by the ubiquitin-proteasome system and phosphorylated in response to cytokine stimulation [12, 13]. Exogenous administration of SOCS proteins can compensate for the degradative loss of endogenous SOCS3 and terminate noxious cytokine signaling during an acute inflammatory response [14].

The pro-inflammatory cytokine IL-6 is a multifunctional cytokine and plays an important role in the development of inflammation, immune process and neuralgia [15]. In the case of inflammation, IL-6, prior to other cytokines, is secreted by innate immune system, and usually for a long duration [16]. Both acute and chronic stress can reportedly increase IL-6 mRNA levels in the rat hypothalamus [17, 18]. Moreover, IL-6 can be immunolocalized in vasopressin-containing magnocellular neurons of the PVN [19].

The anti-inflammatory effect of SOCS3 is well documented in vascular endothelial cells [20], synovial cells [9] and immune cells [14]. Nevertheless, its role in PVN during chronic inflammatory pain remains elusive. In the present study, we investigated that overexpression of SOCS3 in the PVN attenuated chronic inflammatory pain via inhibition of neuronal sensitization. In addition, we also expounded the expression profiles of pro-inflammatory factor IL-6 in PVN in the acute phase of inflammatory pain. Our study may provide insight into a novel molecular mechanistic explication for anti-inflammatory effect of SOCS3, as well as a potential alternative of intervention of chronic inflammatory diseases.

## Materials and methods

### Animals

Healthy adult male Sprague-Dawley (SD) rats (230–250 g, 8 weeks old) were provided by the Experimental Animal Center of Xuzhou Medical University (Xuzhou, China). During the testing session, rats were housed in standard Plexiglas cages and maintained on a standard 12 h light-dark cycle (lights on at 07:00 AM), with constant temperature and humidity (22 °C and 50%, respectively) and ad libitum access to chow and water. All procedures were conducted in accordance with the guidelines as described in the National Institutes of Health’s Guide for the Care and Use of Laboratory Animals (NIH Publication No. 8023, revised 1978) and the International Association for the Study of Pain, and were approved by the Institutional Animal Care and Use Committee at Xuzhou Medical University.

### Reagents

SOCS3 overexpressing and negative control AAV were purchased from BrainVTA (Wuhan, China). IL-6 was purchased from EXCELL (Shanghai, China). IL-6 neutralising antibody (IL-6 NA) was purchased from abcam (Cambridge, UK). Alkaline phosphatase-labeled anti-rabbit and anti-goat secondary antibody were purchased from ZSGB-BIO (Beijing, China). Complete Freund’s adjuvant (CFA) was purchased from Sigma–Aldrich (St. Louis., MO, USA). Rabbit and goat IgG isotype control, ACIP/NBT alkaline phosphatase chromogenic reagent kit, Lysate RIPA and the phosphatase inhibitor phenylmethanesulfonyl- fluoride (PMSF) were purchased from Beyotime Institute of Biotechnology (Jiangsu, China). Anti-p-MEK1/2 antibody (9154S) was purchased from Cell Signaling Technology (Danvers, MA, USA). Anti-GAPDH antibody (Cat. No: AC001) was purchased from ABclonal Biotechnology (Woburn, MA, USA).

### Intra-PVN injection

Rats were anesthetized under isoflurane and mounted onto a David Kopf stereotaxic apparatus (Tujunga, CA, USA) with the skull in horizontal position. The skull was exposed with an incision in the scalp along the midline in each rat. The skull surface was cleansed with hydrogen peroxide and rats PVN nucleus determined: ML, ±0.4 mm; AP, − 1.5 mm; DV, -7.8 mm. IL-6 neutralizing antibody (10 ng/μl, 0.3 μl), AAV (1.17 × 10^12^ vg/ml, 0.5 μl) were injected into the PVN with a 0.3 mm microsyringe at a infusion rate of 0.167 μl/min. The needle was left in site for 3 min to allow for solution diffusion. Rats were returned to their home cages until behavioral testing or brain isolation. The position of the cannula track aiming at the PVN was histologically verified for each brain, and rats with incorrect cannulation were excluded from data analysis.

### Western blotting

Fresh brain tissues were isolated on ice and placed in an Eppendorf (EP) tube and stored at the icy environment. The lysate RIPA and the phosphatase inhibitor PMSF were successively added, followed by homogenization and centrifugation at 12000 g for 30 min at 4 °C, with 80 μl supernatant sampled thereafter. The BCA method was employed to detect and trim the protein concentration after lysis. The PVDF membranes (7.0 cm × 2.5 cm) were activated in methanol for 1–5 s, and agitated thrice for 5 min. After blockade for 2 h at room temperature (r/t), the membranes were rinsed in washing buffer, and agitated at 4 °C overnight, followed by placement at r/t for 30 min. After addition of Washing buffer solution and triplicate agitation for 5 min, the membranes were incubated with alkaline phosphatase-labeled secondary antibody at constant temperature for 2 h and rinsed thrice with washing buffer for 5 min. Protein bands were visualized using the BCIP/NBT Alkaline Phosphatase Color Development Kit and quantified using ImageJ software.

### Immunofluorescent staining

The PVN region of the hypothalamus was sliced at 30 μl thick with a cryostat. Tissue sections were rinsed with Tris Buffered Saline (TBS) twice for 10 min. The brain slices were incubated with primary antibody dissolved in TBS for 24 h at 4 °C and rinsed thrice for 5 min with TBS. Secondary antibody was added with fresh membrane with aluminium foil covered and incubated at r/t for 2 h and rinsed thrice with TBS for 10 min. The tissue sections were visualized with a Leica confocal microscope.

### Paw withdrawal thermal latency (PWTL)

Rats were placed on a clean glass plate with r/t and allowed for acclimation. Pain tester laser intensity was adopted to assess thermal nociceptive responses. PWTL was defined as the time for the onset of paw lifting, with the heat source powered off thereafter. The test was repeated four times at an interval of 5–10 min.

### Paw withdrawal mechanical threshold (PWMT)

Rats were placed in a cage at r/t for acclimation for 30 min. The plantar surfaces of the hind paws were stimulated from the 10 g Von Frey filament. The presence of paw lifting, avoidance or claudication in 10 s was defined as the positive response and absence of reaction was regarded as negative. The same Von Frey filament assessment was repeated at an interval of 3–5 min. In the case of 3 positive reactions, adjacent low-intensity stimulation was employed, and in the event of 3 negative reactions, increment of high-intensity stimulation was adopted. In brief, five assessments were undertaken in each rat.

### Statistical analysis

Data were expressed as mean ± SEM. Statistical analysis was performed using SPSS 19.0 (IBM, Armonk, NY, USA). All statistical analyses were two-tailed comparisons. All data met the assumptions of the statistical tests used. The data were analyzed using unpaired *t*-test, one-way ANOVA, and two-way ANOVA. *P* < 0.05 was considered statistically significant.

## Results

### Expression of IL-6 and SOCS3 in PVN in rat model of inflammatory pain induced by subcutaneous injection of CFA

**Fig. 1:**
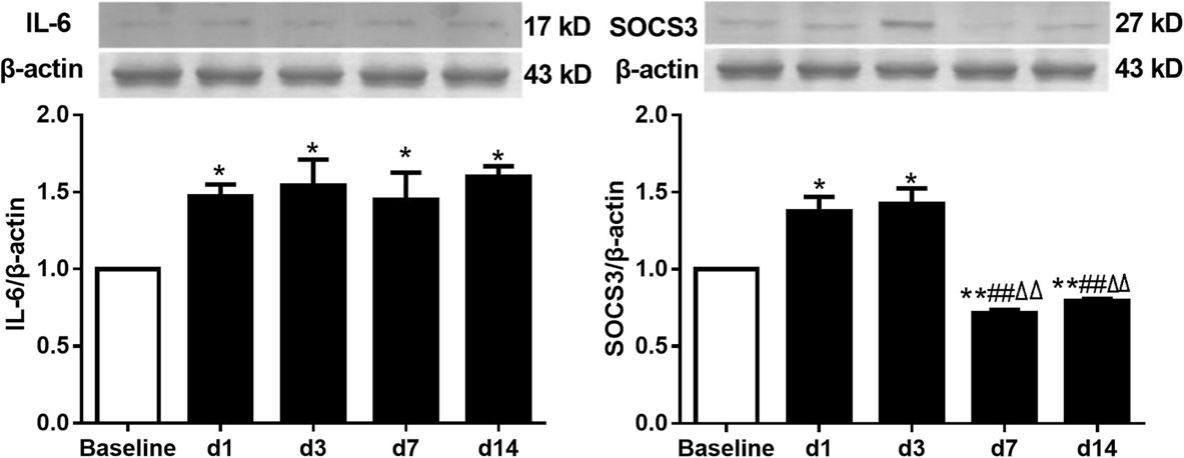
The expression of IL-6 and SOCS3 in PVN in rat model of inflammatory pain induced by subcutaneous injection of CFA. (**a**) (**b**) represent the expression of IL-6 and SOCS3 protein in PVN on days 1, 3, 7, 14 after CFA administration, respectively (one-way ANOVA, *n* = 6). ^**^P < 0.01 vs. baseline, ^##^*P* < 0.01 vs. day 1, ^△△^*P* < 0.01 vs. day 3

Subcutaneous injection of CFA into the hind paws of rat established a canonical model of chronic inflammatory pain. Western blotting analysis showed that the expression of IL-6 was significantly increased in both acute and chronic phases of inflammatory pain versus baseline (Fig. 1a, *P* < 0.05). In addition, the expression of SOCS3 protein was increased on day 1 and 3 after CFA administration (Fig. 1b, *P* < 0.05), and was relatively decreased on day 7 and 14 after CFA administration (Fig. 1b, *P* < 0.01).

### The effects of intra-PVN injection of IL-6 neutralizing antibody on the inflammatory pain in acute phase in rats

**Fig. 2:**
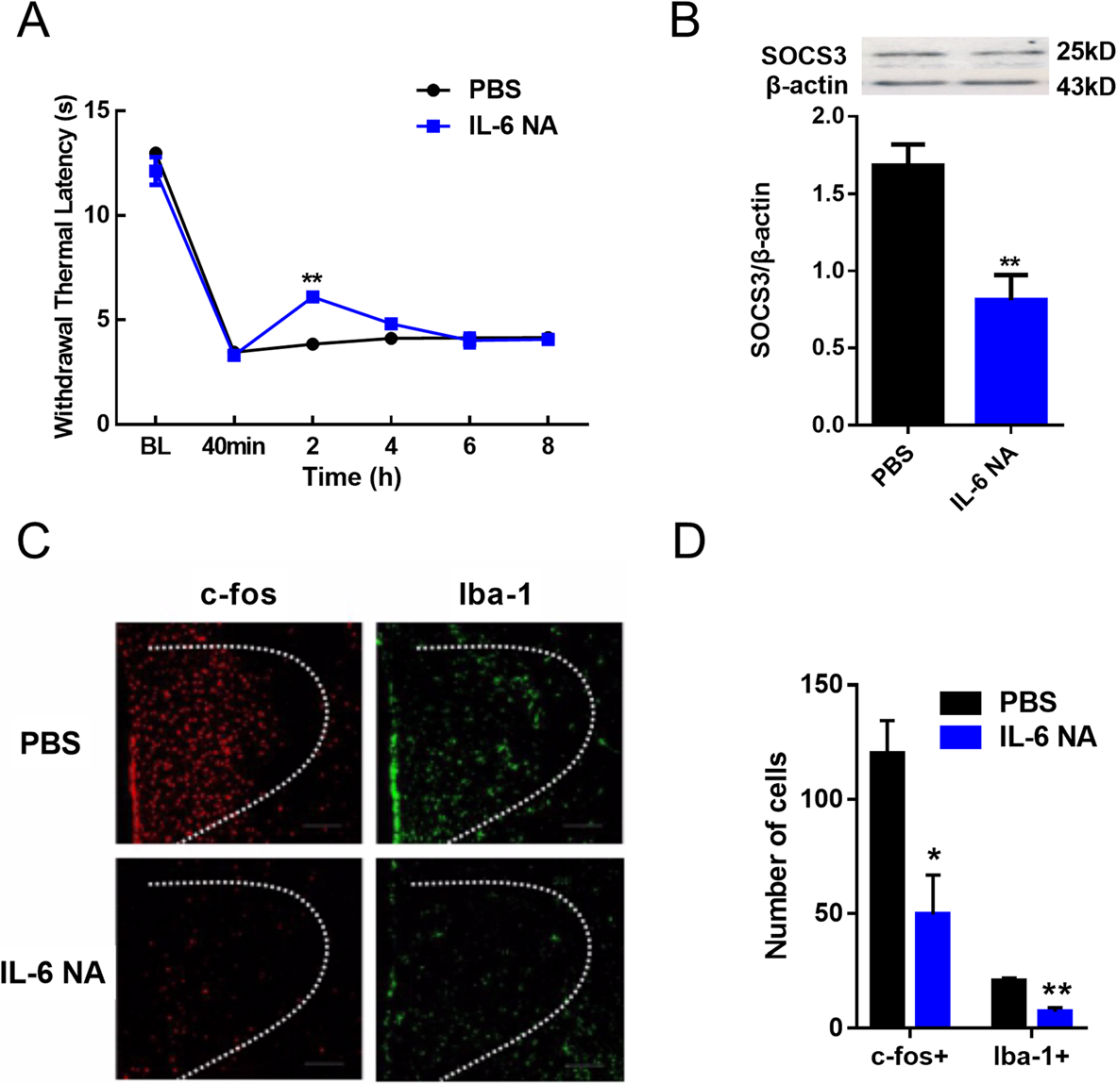
The effects of intra-PVN injection of IL-6 neutralizing antibody on inflammatory pain in the acute phase in rats. (**a**) The effects of intra-PVN injection of IL-6 neutralizing antibody on TWL (one-way ANOVA, n = 6). (**b**) The expression of SOCS3 protein in PVN on day 7 after CFA administration (unpaired *t*-test, n = 6). (**c**)(**d**) Representative images of c-fos and Iba-1. IL-6 NA group presented a significant decrease in the number of c-fos-positive and Iba-1-positive neurons in PVN versus PBS group (unpaired *t*-test, n = 6). ^*^P < 0.5 vs. PBS group

To investigate the function of IL-6 in the acute phase of inflammatory pain, rats underwent subcutaneous injection of CFA, and 1 day thereafter micro-injection of IL-6 neutralizing antibody into PVN, with thermal pain threshold recorded at post-micro-injection 40 min, 2 h, 4 h, 6 h and 8 h, respectively. As shown in Fig. 2a, thermal hyperalgesia was relieved subsequent to the injection of IL-6 neutralizing antibody. Moreover, Western blotting analysis revealed the significant downregulation of SOCS3 expression versus PBS group (*P* < 0.01; Fig. 2b). Microglial activation and neuronal excitability were evaluated by Iba-1 and c-fos immunofluorescence labeling, respectively. IL-6 neutralizing antibody group presented a notable decrease in Iba-1 labeling in PVN (Fig. 2c). Further, the population of c-fos-positive cells presented with a marked decrease versus PBS group (*P* < 0.01; Fig. 2d). These results demonstrate that inflammatory pain in the acute phase is associated with microglial activation and neuronal sensitization via IL-6 in the PVN.

### The effects of intra-PVN injection of AAV overexpressing SOCS3 (SOCS3-AAV) on inflammatory pain in the chronic phase in rats

**Fig. 3:**
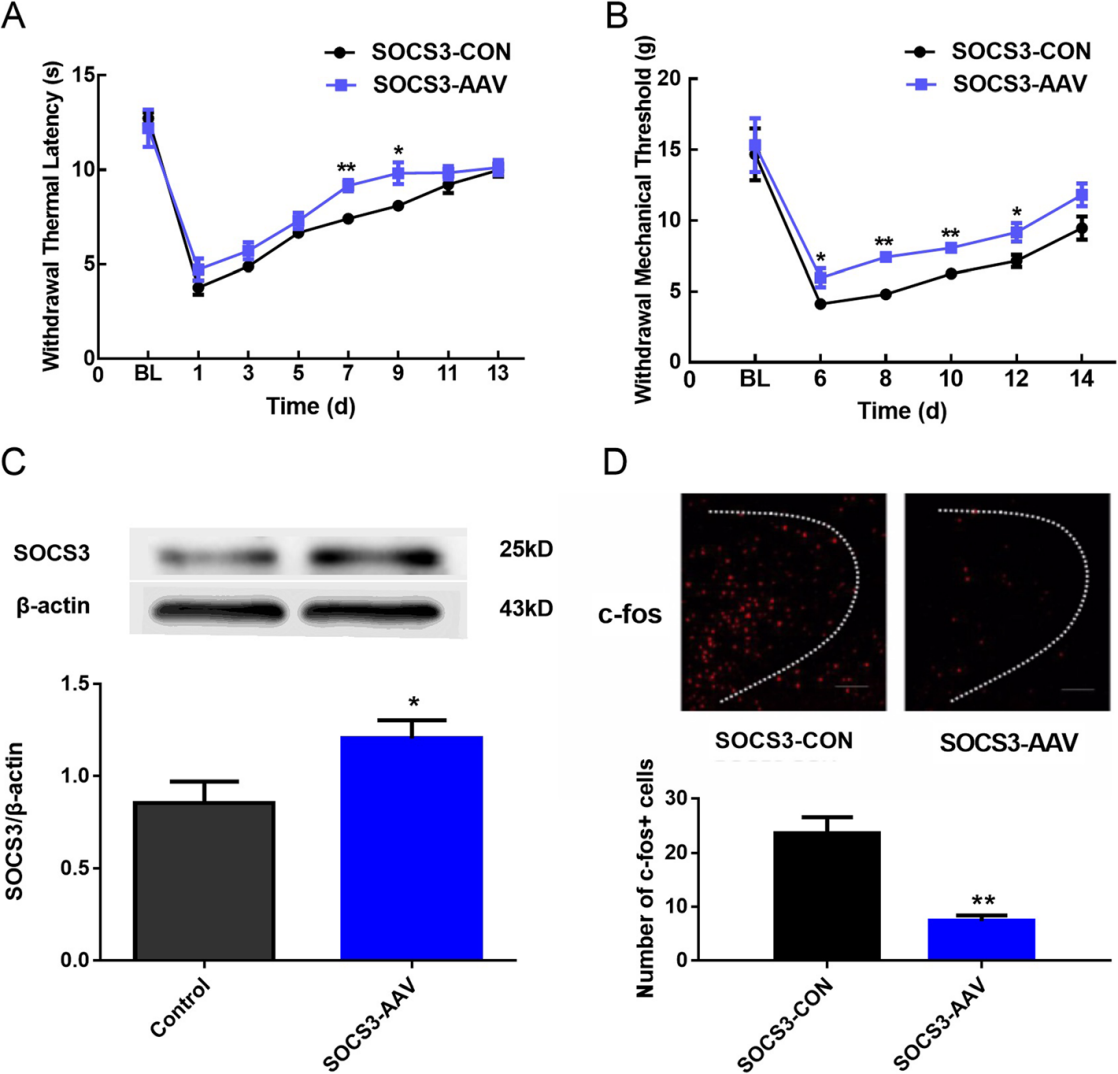
The effects of intra-PVN injection of AAV overexpressing SOCS3 (SOCS3-AAV) on inflammatory pain in the chronic phase in rats. (**a**)(**b**) The effects of intra-PVN injection of SOCS3-AAV on TWL and MWT in rats receiving CFA. (two-way ANOVA, n = 6). (**c**) The expression of SOCS3 in PVN in rats receiving CFA on day 7 after intra-PVN injection of SOCS3-AAV and SOCS3-CON (unpaired *t*-test, n = 6). (**d**) Representative images of c-fos in the PVN. SOCS3-AAV group presented a significant decrease in the number of c-fos-positive neurons in PVN versus SOCS3-CON group (unpaired *t*-test, *n* = 8). ^*^P < 0.5 vs. SOCS3-CON group

Twenty one days after intra-PVN injection of AAV overexpressing SOCS3, thermal pain threshold was recorded on day 1, 3, 5, 7, 9, 11 and 13, and mechanical allodynia threshold was recorded on day 6, 8, 10, 12 and 14 after CFA administration. As depicted in Fig. 3a and Fig. 3b, thermal hyperalgesia and mechanical allodynia were significantly increased versus control group (*P* < 0.01), and reached the peak between day 7 and 9. In parallel, Western blotting analysis revealed the expression of SOCS3 protein in PVN was remarkably increased on day 7 (Fig. 3c). Immunofluorescent assay suggested a decrease in the population of c-fos-positive cells in the PVN in the SOCS3-AAV group versus the control group (Fig. 3d). These results indicate that SOCS3 signaling plays an important role in anti-inflammatory effect.

## Discussion

Pain is meditated by multisynaptic pathways which are triggered in the periphery and subsequently processed at multiple sites in the CNS. Long-term potentiation of synaptic transmission increased neuronal response to peripheral nociceptive stimulation, leading to central sensitization [21]. Unremitting excitation of primary nociceptive neurons induces chronic pain [3]. The PVN, especially the dorsal parvocellular subdivisions, has extensive descending projections to reticular and autonomic portions of the brainstem and spinal cord, and receives heavy projections from nociceptive pathways. Nonetheless, nociception is a robust stimulus of the PVN and HPA axis [22]. Acute inflammation stimulates the HPA stress axis, resulting in activation of the CRF neurons within the medial and dorsal parvocellular divisions of the PVN [23]. CRF mRNA in the PVN is reportedly elevated in active colitis [24]. These findings indicate that neuronal activation in PVN participates in the regulation of inflammation. In the present study, we explored the expression of IL-6 and SOCS3 protein in the PVN during inflammatory insult. Paradoxically, the IL-6 expression was overexpressed throughout inflammation, whereas the expression of SOCS3 was increased in the acute phase but was decreased in the chronic phase. Intra-PVN injection of IL-6 NA attenuated thermal hyperalgesia in the acute phase by inhibiting microglia activation and neuronal sensitization. Furthermore, Intra-PVN injection of AAV overexpressing SOCS3 prevented the progression of inflammation by reducing neuronal sensitization in the chronic phase. Our findings confirmed that overexpression of SOCS3 had a protective effect in chronic inflammatory pain, which may be implicated in central sensitization.

Glial activation results in pathological inflammatory responses such as neuronal sensitization, neurotoxicity and chronic inflammation [25]. Microglial activation is an early event in CNS inflammation, with large amount of IL-6 released in response to peripheral input stimulus [26]. By acting on γ-aminobutyric acid (GABA) receptors of sensitive neurons, IL-6 locally secreted in the PVN decreases the sensitivity of tonic inhibition of GABAergic fiber to promote neuronal sensitization, thereafter resulting in pathological inflammatory response. In addition, IL-6 phosphorylated STAT3 to promote inflammatory response in the course of inflammation. As a target of STAT3 transcriptional activation, SOCS3 is robustly increased in the hypothalamus due to acute stress [27]. In our study, we observed that administration of IL-6 neutralizing antibody in PVN relieved CFA-induced thermal hyperalgesia. This effect was related to blockade of IL-6-mediated inflammatory signaling, including inhibition of neuronal sensitization and reduction of microglial activation.

SOCS3 can be induced by JAK/STAT signaling [10], exchange protein directly activated by cAMP (Epac) [20] and mitogen-activated protein kinase (MAPK) [28], and is involved in immunity [29], incretion [30] and neural development [31]. Synergistic actions of IL-6, granulocyte colony-stimulating factor (G-CSF), monocyte chemotactic protein (MCP)-1 and microphage infectivity potentiator (MIP) drive inflammatory response in the absence of SOCS3 [32]. In addition, deletion of SOCS3 drastically increases axon re-growth in the CNS neurons and activates inflammatory responses [33] and the activation of STAT3/Th17 pathway is essential to the induction of elevated inflammatory cytokines and the increase of superoxide release in the lung [34]. However, exogenous SOCS3 had a positive effect on airway inflammation by suppression of Th17-mediated neutrophil recruitment [35]. Such evidence indicates SOCS3 might be a molecular switch of inflammation. Prior reports indicated that SOCS3 was increased at both sites of acute and chronic inflammation and was at a higher level in acute than chronic inflammation [36]. Intriguingly, in our study, we evidenced that the expression of SOCS3 protein was relatively decreased during chronic phase of inflammatory pain, whereas IL-6 protein expression was still at a plateau. Down-regulated expression of SOCS3 blunts the inhibition to STAT3 pathway. Therefore, we speculated that the SOCS3 downregulation participates in the pathological process of chronic inflammatory pain. Our results revealed that adenoviral gene transfer of cytokine signal inhibitor SOCS3 efficiently suppressed the process of chronic inflammatory pain and this effect was related to suppression of neuronal activation. However, the employment of adenovirus vectors for therapy for inflammation is controversial, since adenovirus vector-mediated gene transduction is transient, and local injection of adenovirus sometimes induces inflammation [37, 38]. In addition, the mechanism underlying the downregulated SOCS3 expression in chronic phase has not been fully explicated. A more compelling interpretation of SOCS3-mediated intracellular signal regulation in CNS warrants further investigation.

## Conclusions

In conclusion, we demonstrate the expression of intracellular signaling protein SOCS3 in the PVN in the acute and chronic phases of inflammatory pain, respectively. Blockade of IL-6 inflammatory signaling relieves inflammatory pain in the acute phase, accompanied by inhibition of neuronal sensitization and reduction of microglial activation. Overexpression of SOCS3 attenuates chronic inflammatory pain and this effect is related to inhibition of neuronal sensitization. Our study may shed new light on the investigation of molecular mechanism for anti-inflammatory effect of SOCS3 and contribute to intervention of chronic inflammatory diseases.

## Supplementary information

**Additional file 1.** The immunofluorescence staining assay of anti-goat (left) and anti-rabbit (right) isotope control antibodies revealed no non-specific staining.

## Data Availability

The data to support the findings of this study are available from the corresponding author upon request.

## Abbreviations

CFA: Complete Freund’s adjuvant
CNS: Central nervous system
Epac: Exchange protein directly activated by cAMP
GABA: γ-aminobutyric acid
IL-1β: Interleukin-1β
IL-6 NA: IL-6 neutralizing antibody
IL-6: Interleukin 6
JAK/STAT: Janus kinase/signal transducer and activator of transcription 3
PVN: Paraventricular nucleus
SOCS3 AAV: SOCS3 adeno-associated virus
SOCS3: Suppresser of cytokine signaling 3
TNF-α: Tumor necrosis factor α
MAPK: Mitogen-activated protein kinase
CSF: Granu1ocyte colony-stimu1ating factor.
MCP1: Monocyte chemotactic protein 1
MIP: Microphage infectivity potentiator
CRF: Corticotropin releasing factor
TBS: Tris Buffered Saline
EP: Eppendorf
PMSF: Phenylmethanesulfonyl fluoride
