# Supplementary figures and images for: The role of SOCS3 in the hypothalamic paraventricular nucleus in rat model of inflammatory pain
Source: J Inflamm (Lond). 2020 Feb 27;17:12. doi: 10.1186/s12950-020-00241-9 (PMC7047413; doi:10.1186/s12950-020-00241-9)

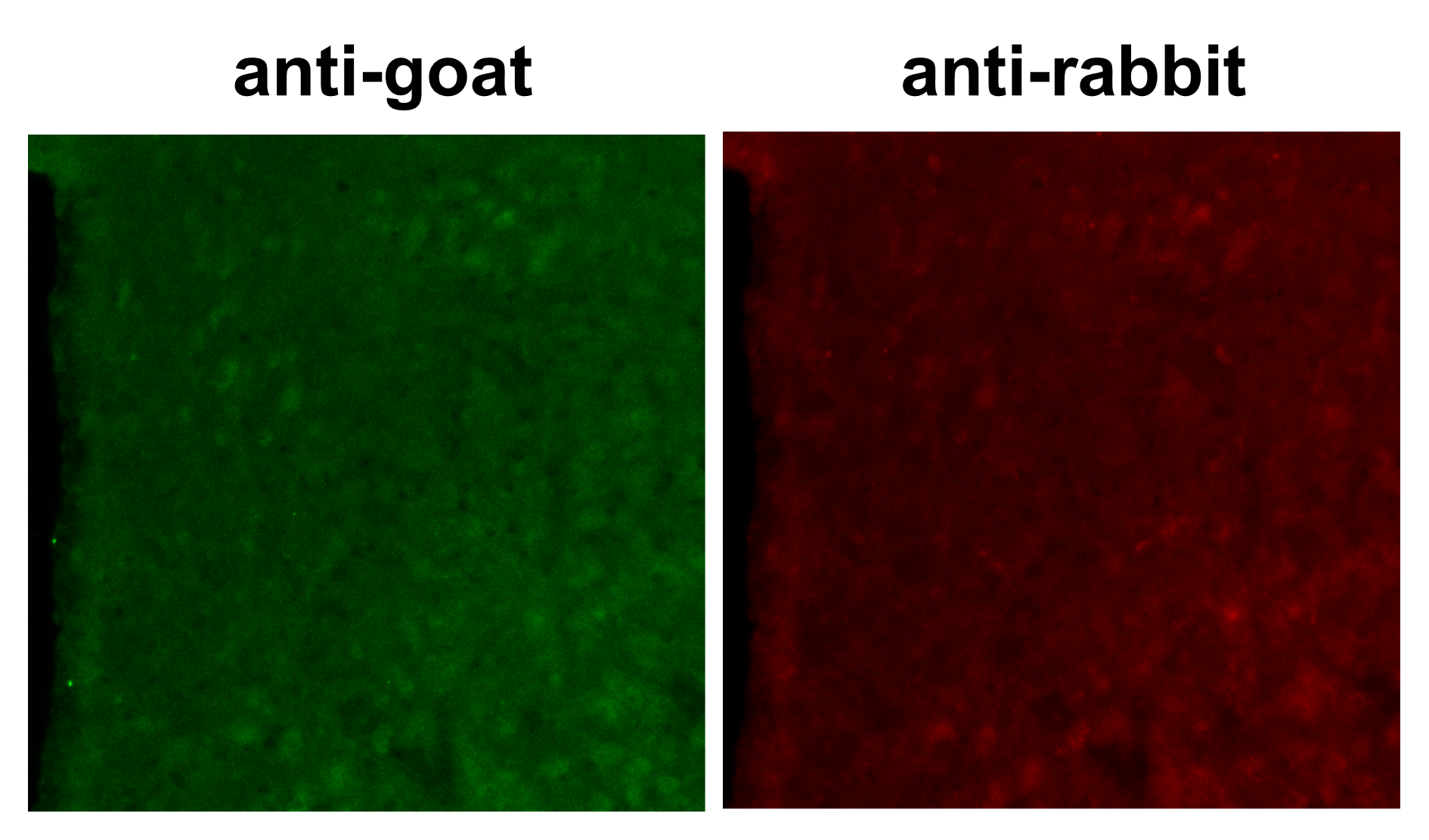

Supplement: Supplementary file 1 — Additional file 1. The immunofluorescence staining assay of anti-goat (left) and anti-rabbit (right) isotope control antibodies revealed no non-specific staining. [file 12950_2020_241_MOESM1_ESM.tif]
